# Doença cística da adventícia na veia basílica: relato de caso

**DOI:** 10.1590/1677-5449.002616

**Published:** 2016

**Authors:** Rafael Sampaio Vasconcelos, Cesar Augusto Cherubim, Felipe Mavignier Pereira França, Eduardo de Lucca D'allacqua, Marcelo Bellini Dalio, Edwaldo Edner Joviliano

**Affiliations:** 1 Universidade de São Paulo – USP, Faculdade de Medicina de Ribeirão Preto, Hospital das Clínicas, Departamento de Cirurgia e Anatomia, Divisão de Cirurgia Vascular e Endovascular, Ribeirão Preto, SP, Brasil.

**Keywords:** doença cística da adventícia, veia basílica, angiorressonância magnética

## Abstract

A doença cística da adventícia é uma entidade rara que acomete principalmente a artéria poplítea. A ocorrência em veias é muito rara, e sua etiologia é desconhecida. Clinicamente, apresenta-se como isquemia, trombose ou dor a depender do território acometido. Apresentamos o caso de um paciente masculino jovem referindo nódulo no braço esquerdo. A angiorressonância magnética do membro mostrou lesão cística em contato com a veia basílica, com conteúdo homogêneo e sem realce pós-contraste. Foi realizada ressecção da lesão em bloco com o segmento venoso envolvido. O estudo anatomopatológico foi sugestivo de cisto de adventícia de veia basílica.

## INTRODUÇÃO

A doença cística da adventícia é uma entidade rara^1^ que se caracteriza por degeneração cística da camada adventícia, com presença de conteúdo mucoide^2^^,^ 3. A maioria dos relatos na literatura descreve casos em artérias^4^^,^ 5. Em veias, a doença cística da adventícia é extremamente rara^1^. Não encontramos relatos de casos de doença cística da adventícia em veias do membro superior. O objetivo deste artigo é relatar um caso raro de doença cística da adventícia em veia basílica tratado com sucesso por ressecção da lesão em bloco com segmento venoso acometido. O paciente autorizou a publicação do caso por meio de termo de consentimento informado.

## DESCRIÇÃO DO CASO

**Figura 1:**
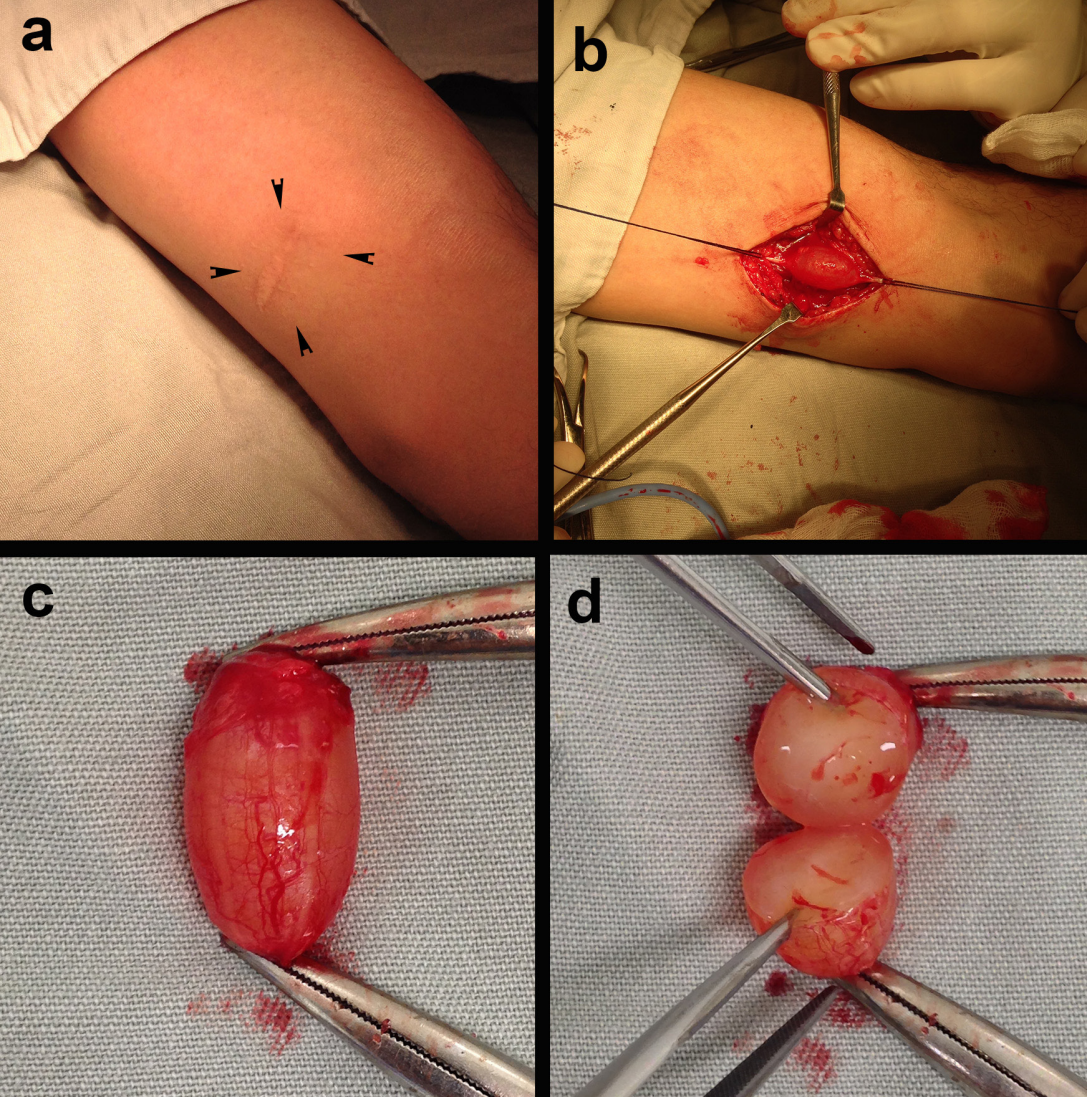
Imagens mostrando aspecto pré-operatório (a); lesão cística dissecada e firmemente aderida à veia basílica, que foi reparada com fios de algodão (b); lesão ressecada (c) e lesão seccionada evidenciando seu conteúdo (d).

**Figura 2:**
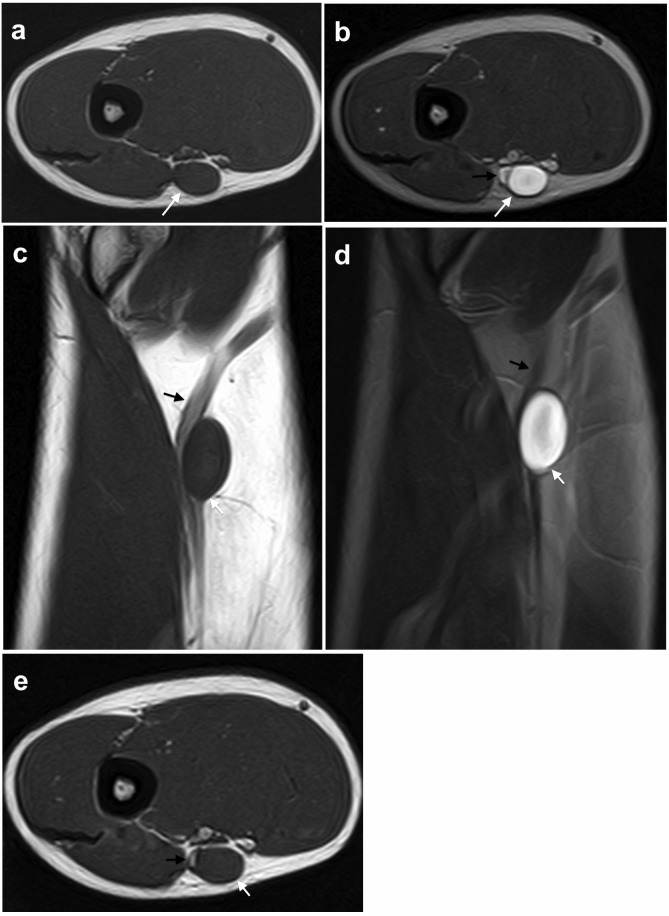
Angiorressonância magnética do membro superior esquerdo evidenciando lesão cística de paredes lisas e conteúdo homogêneo (setas brancas) adjacente e em contato com a parede da veia basílica (setas pretas), em cortes axial T1 (a), axial T2 (b), coronal T1 (c) e coronal T2 (d). As imagens obtidas após a administração de contraste em cortes coronal T1 (c) e axial T1 (e) não mostraram realce da lesão.

Paciente masculino, 34 anos, branco, relatava abaulamento na face medial do braço esquerdo com dois anos de evolução. Procurou assistência em serviço de assistência primária, onde foi levantada a hipótese de lipoma de tecido celular subcutâneo e realizada tentativa de ressecção sob anestesia local. Durante o procedimento, foi observada íntima relação da lesão com a veia basílica. Optou-se então por abortar o procedimento e encaminhar o paciente ao nosso serviço. Na consulta inicial, referia dor local. Ao exame físico, apresentava nódulo bem delimitado de consistência fibroelástica de aproximadamente 2,5 x 3,0 cm na face medial do braço esquerdo, com dor leve à palpação e sem sinais flogísticos (Figura 1a). Apresentava cicatriz da incisão prévia com bom aspecto. Realizamos a investigação com angiorressonância magnética do membro superior, que evidenciou lesão cística em contato com a parede da veia basílica, causando compressão do vaso. A lesão tinha paredes lisas, conteúdo homogêneo e sem realce pós-contraste (Figura 2). Não havia trombose da veia basílica, e as demais veias do membro estavam bem contrastadas.

O paciente foi submetido a abordagem cirúrgica com dissecção da lesão e controle da veia basílica. O cisto apresentava pouca aderência aos planos adjacentes e estava intimamente relacionado à veia basílica, sem plano de clivagem entre a lesão e a veia (Figura 1b). Optou-se pela ressecção da lesão juntamente com o seguimento de veia basílica envolvido. Os cotos da veia basílica foram tratados com ligadura simples. O estudo anatomopatológico macroscópico evidenciou cisto de paredes finas, formado por tecido conjuntivo denso e preenchido por material coloide (Figuras 2c e 2d). A análise microscópica revelou conteúdo paucicelular formado por fibroblastos maduros e sem atipias, fibras frouxas de tecido conjuntivo e capilares esparsos. Esses achados confirmaram a hipótese de cisto de camada adventícia da veia basílica. O paciente evolui sem queixas e complicações.

## DISCUSSÃO

A doença cística da adventícia é uma entidade rara e de etiologia desconhecida^1^. Quatro teorias tentam explicar sua origem: teoria ganglionar (células sinoviais implantadas na adventícia), teoria traumática (degeneração devido a traumas locais), teoria do desenvolvimento (implantes durante embriogênese) e teoria da doença sistêmica (secundária a doença sistêmica do tecido conjuntivo)^1^^,^ 6. A maioria dos relatos na literatura descreve casos em artérias^4^. Em veias, a doença cística da adventícia é extremamente rara^6^^,^ 7. Francis et al. descreveram três casos envolvendo veias da região ilíaco-femoral, onde ela é mais comumente encontrada^8^. Numa revisão recente de Desy & Spinner, nenhum relato de caso dessa condição foi descrito em veias dos membros superiores^1^. Devido a essa raridade, sua suspeita clínica é geralmente tardia.

A apresentação clínica dessa condição é variável e depende do território acometido^9^^-^ 12. Em artérias, ela pode se manifestar clinicamente como um quadro de isquemia de membro ou dor por compressão local^13^. Em veias, manifesta-se como dor local ou trombose venosa^7^. Como no presente caso, pode ser confundida com causas mais comuns de nódulos no tecido subcutâneo, como lipoma, adenomegalia, cisto sebáceo e fibroma^1^. A suspeita diagnóstica da doença cística da adventícia em veias deve ser considerada em lesões nodulares na topografia de trajetos venosos. A confirmação diagnóstica geralmente requer um exame de imagem. A ultrassonografia, devido ao baixo custo, alta disponibilidade e não necessidade de uso de contraste injetável, é geralmente o primeiro exame a ser realizado. A lesão aparece como nódulo bem definido, de conteúdo anecoide. A angiotomografia computadorizada permite adequada visualização da lesão, mas tem alto custo, necessita de injeção de contraste e emite radiação ionizante. A angiorressonância magnética é o estudo que demonstra melhor definição dos planos anatômicos, ajuda no planejamento cirúrgico e permite diagnóstico diferencial com cisto articular^1^. No entanto, também apresenta alto custo e requer infusão de gadolínio como meio de contraste. No presente caso, optamos pela angiorressonância magnética devido às vantagens acima descritas.

As opções de tratamento incluem seguimento clínico, aspiração percutânea guiada por imagem, angioplastia com e sem stent, ressecção simples do cisto e ressecção do cisto com reconstrução vascular^1^^,^ 14. Em revisão recente, Desy & Spinner descreveram que a modalidade terapêutica mais utilizada é a ressecção da lesão, com ou sem ressecção em bloco de vaso acometido^1^. Descreveram também que após a ressecção, deve ser avaliada a necessidade de realizar reconstrução vascular com veia autóloga ou material sintético. No presente caso, como o paciente apresentava sintomas de compressão local, foi proposta a ressecção da lesão. Durante o procedimento, foi observado que os planos anatômicos se apresentavam bem definidos, possibilitando a dissecção sem dificuldade. No entanto, devido a sua íntima relação com a veia basílica, não foi possível a ressecção individual do cisto. Como o paciente apresentava perviedade do sistema venoso superficial e profundo no membro, optamos pela ressecção em bloco, juntamente com a veia basílica. Como há geralmente grande reserva funcional na drenagem venosa do membro superior, a veia basílica pode ser ressecada sem causar sequelas. Outra modalidade terapêutica descrita é a aspiração do conteúdo do cisto guiada por ultrassonografia^15^. Esse método terapêutico tem a vantagem de ser menos invasivo, mas não se aplica a todos os casos. O conteúdo do cisto é geralmente espesso e nem sempre pode ser aspirado com agulha^16^. O tratamento endovascular não tem se mostrado efetivo nessa afecção^17^, e não encontramos relatos dessa modalidade de tratamento em veias.

## CONCLUSÃO

A doença cística da adventícia na veia basílica é uma condição rara que se apresenta como nódulo na região medial do braço. Deve ser considerada no diagnóstico diferencial de lesões nodulares nessa região. A ressecção da lesão em bloco, juntamente com a veia basílica, apresentou bom resultado.
