# Supplementary material for: Doença cística da adventícia na veia basílica: relato de caso
Source: J Vasc Bras. 2016 Jul-Sep;15(3):245–9. [Article in Portuguese] doi: 10.1590/1677-5449.002616 (PMC5829763; doi:10.1590/1677-5449.002616)
Supplement: Translation

# Translation: Basilic vein cystic adventitial disease: case report

**Authors:** Rafael Sampaio Vasconcelos, Cesar Augusto Cherubim, Felipe Mavignier Pereira França, Eduardo de Lucca D'allacqua, Marcelo Bellini Dalio, Edwaldo Edner Joviliano

**Affiliations:** 1 Universidade de São Paulo – USP, Faculdade de Medicina de Ribeirão Preto, Hospital das Clínicas, Departamento de Cirurgia e Anatomia, Divisão de Cirurgia Vascular e Endovascular, Ribeirão Preto, SP, Brazil.

**Keywords:** cystic adventitial disease, basilic vein, magnetic resonance angiography

## Abstract

Cystic adventitial disease is a rare entity that most often involves the popliteal artery. It rarely occurs in veins. Its etiology is unknown. Clinically, it presents with ischemia, thrombosis or pain, depending on the vessel affected. Here we present the case of a young male with a nodule in the left arm. Magnetic resonance angiography showed a cystic lesion in contact with the basilic vein, with homogenous content without post-contrast enhancement. The lesion was resected *en bloc* together with the venous segment involved. The results of microscopic analysis were suggestive of basilic vein cystic adventitial disease.

## INTRODUCTION

Cystic adventitial disease is a rare entity^1^ that is characterized by cystic degeneration of the adventitial layer, with mucoid cystic contents.^2^^,^ 3 The majority of reports in the literature describe cases in arteries.^4^^,^ 5 Cystic adventitial disease of veins is extremely rare.^1^ We were unable to locate any reports of cases of cystic adventitial disease involving veins of the upper limbs. The objective of this article is to report on a rare case of cystic adventitial disease of the basilic vein that was successfully treated by resection *en bloc* together with the segment of vein involved. The patient authorized publication of the case via signature of an Informed Consent Form.

## CASE DESCRIPTION

A 34-year-old, white, male patient presented complaining of swelling on the medial surface of his left arm with onset 2 years previously. He sought care at a primary care service, which raised a hypothesis of subcutaneous tissue lipoma and made an attempt at resection under local anesthesia. During the procedure, it was observed that the lesion was intimately related to the basilic vein and the decision was taken to abort the attempt and refer the patient to our vascular service. At the initial consultation the patient complained of localized pain. A physical examination detected a well-defined nodule with fibroeleastic consistency on the medial surface of the left arm, measuring approximately 2.5 x 3.0 cm, provoking mild pain on palpation and with no signs of inflammation (Figure 1a). The wound left by the prior incision was visible and had a satisfactory appearance. We conducted an investigation using magnetic resonance angiography of the upper limb, which showed a cystic lesion in contact with the wall of the basilic vein, causing compression of the vessel. The lesion was smooth-walled, with homogenous content and was not highlighted after administration of contrast (Figure 2). There was no thrombosis of the basilic vein and the other veins in the limb were clearly visible with contrast.

**Figure 1:** Images showing the preoperative appearance (a); the cystic lesion dissected and firmly adhering to the basilic vein, which was repaired with cotton thread (b); the resected lesion (c); and the lesion sectioned, showing its content (d).

**Figure 2:** Magnetic resonance angiography of the left upper limb showing a cystic lesion with smooth walls and homogenous content (white arrows) adjacent to and in contact with the wall of the basilic vein (black arrows), in T1 axial (a), T2 axial (b), T1 coronal (c), and T2 coronal (d) views. Images acquired after administration of contrast, in T1 coronal (c) and T1 axial (e) views did not show contrast highlighting the lesion.

The patient was treated surgically, with dissection of the lesion and control of the basilic vein. The cyst did not exhibit significant adherence to the adjacent tissue layers, but was intimately related to the basilic vein, with no plane of cleavage between the lesion and the vein (Figure 1b). It was decided to resect the lesion together with the segment of the basilic vein that was involved. The stumps of the basilic vein were treated with simple ligature. Macroscopic anatomopathological examination revealed a cyst with thin walls formed by dense connective tissue and filled with colloid material (Figures 2c and 2d). A microscopic analysis revealed contents with sparse distribution of cells, consisting of mature fibroblasts free from atypia, loose connective tissue fibers and sparse capillaries. These findings confirmed the hypothesis of a cyst of the adventitial layer of the basilic vein. The patient recovered with no complaints or complications.

## DISCUSSION

Cystic adventitial disease is a rare entity of unknown etiology.^1^ Four theories have been proposed in attempts to explain its origins: the ganglion theory (synovial cells implanted in the adventitial layer), the traumatic theory (degeneration caused by local traumas), the developmental theory (implants occur during embryogenesis), and the systemic disease theory (secondary to systemic connective tissue disease).^1^^,^ 6 The majority of the reports in the literature describe cases in arteries.^4^ Cystic adventitial disease involving veins is extremely rare.^6^^,^ 7 Francis et al. described three cases involving veins in the iliofemoral region, where the condition is most commonly found.^8^ A recent review by Desy & Spinner did not include any case reports in which this condition was described in veins of the upper limbs.^1^ This degree of rarity means that clinical suspicion is generally late.

The condition’s clinical presentation varies depending on the territory involved.^9^^-^ 12 In arteries, it may manifest clinically with ischemia of a limb or pain caused by localized compression.^13^ In veins, it manifests as local pain or venous thrombosis.^7^ As happened in the case described here, it may be confused with more common causes of nodules in subcutaneous tissues, such as lipomas, adenomegaly, sebaceous cysts, and fibromas.^1^ Diagnostic suspicion of cystic adventitial disease in veins should by aroused when nodular lesions are found along the paths of veins. Diagnostic confirmation will generally require an imaging exam. In view of the low cost, wide availability and no need for injected contrast, ultrasonography is generally the first examination used. The lesion will appear as a well-defined nodule, with anechoic content. Computed tomography angiography offers adequate visualization of the lesion, but is expensive, requires injection of contrast and emits ionizing radiation. Magnetic resonance angiography is an examination that offers better definition of anatomic planes, facilitates surgical planning and enables differential diagnosis to rule out articular cysts.^1^ However, it is also expensive and requires infusion of gadolinium as a contrast medium. In the case described here, we used magnetic resonance angiography because of the advantages mentioned above.

Treatment options include clinical follow-up, percutaneous aspiration guided by imaging, angioplasty, with and without stents, simple resection of the cyst and resection of the cyst with vascular reconstruction.^1^^,^ 14 In their recent review, Desy & Spinner stated that the most common treatment approach is resection of the lesion, with or without resection en bloc with the vessel involved.^1^ They also stated that after resection, the need for vascular reconstruction with autologous vein or synthetic material should be evaluated. Since the patient in the present case exhibited symptoms of localized compression, resection of the lesion was proposed. During the procedure it was found that the anatomic planes were well-defined, making dissection possible without difficulty. However, because of the cyst’s intimate relation with the basilic vein, it was not possible to resect it without affecting the vein. Since the patient’s superficial and deep vein systems were patent, we opted for resection en bloc with the basilic vein. There is generally a considerable functional reserve of venous drainage in the upper limb and so the basilic vein can be resected without causing sequelae. Another treatment method that has been described is aspiration of the content of the cyst under ultrasonographic guidance.^15^ This method offers the advantage of being less invasive, but is not applicable to all cases. The content of the cyst is generally viscous and cannot always be aspirated with a needle.^16^ Endovascular treatment has not proven effective with this condition,^17^ and we did not find reports of this treatment method for adventitial cysts of veins.

## CONCLUSIONS

Cystic adventitial disease of the basilic vein is a rare condition that presents as a nodule in the medial part of the arm. It should be considered when conducting differential diagnosis of nodular lesions in this region. Resection of the lesion en bloc with the basilic vein segment involved produced good results.
